# Machine Learning of Serum Metabolic Patterns Encodes Asymptomatic SARS-CoV-2 Infection

**DOI:** 10.3389/fchem.2021.746134

**Published:** 2021-10-01

**Authors:** Qiongqiong Wan, Moran Chen, Zheng Zhang, Yu Yuan, Hao Wang, Yanhong Hao, Wenjing Nie, Liang Wu, Suming Chen

**Affiliations:** ^1^ The Institute for Advanced Studies, Wuhan University, Wuhan, China; ^2^ School of Life Sciences, Central China Normal University, Wuhan, China; ^3^ Hubei Key Laboratory of Environmental Health (Incubating), Department of Occupational and Environmental Health, Huazhong University of Science and Technology, Wuhan, China

**Keywords:** COVID-19, MALDI-MS, machine learning, diagnosis, metabolism

## Abstract

Asymptomatic COVID-19 has become one of the biggest challenges for controlling the spread of the SARS-CoV-2. Diagnosis of asymptomatic COVID-19 mainly depends on quantitative reverse transcription PCR (qRT-PCR), which is typically time-consuming and requires expensive reagents. The application is limited in countries that lack sufficient resources to handle large-scale assay during the COVID-19 outbreak. Here, we demonstrated a new approach to detect the asymptomatic SARS-CoV-2 infection using serum metabolic patterns combined with ensemble learning. The direct patterns of metabolites and lipids were extracted by matrix-assisted laser desorption/ionization mass spectrometry (MALDI-MS) within 1 s with simple sample preparation. A new ensemble learning model was developed using stacking strategy with a new voting algorithm. This approach was validated in a large cohort of 274 samples (92 asymptomatic COVID-19 and 182 healthy control), and provided the high accuracy of 93.4%, with only 5% false negative and 7% false positive rates. We also identified a biomarker panel of ten metabolites and lipids, as well as the altered metabolic pathways during asymptomatic SARS-CoV-2 Infection. The proposed rapid and low-cost approach holds promise to apply in the large-scale asymptomatic COVID-19 screening.

## Introduction

The coronavirus disease 2019 (COVID-19) pandemic caused by the severe acute respiratory syndrome coronavirus 2 (SARS-CoV-2) presents an unprecedented threat to global public health (Wu et al., 2020; Zhou et al., 2020). As of 29th April 2021, the SARS-CoV-2 has infected 148,999,876 people around the world, and the death toll has risen to 3,140,115. Although vaccination is in progress, the shortage of vaccines and SARS-CoV-2 variants will make this disease threatening over a considerable period of time. The daily new cases is still more than 669 thousands. Most patients with SARS-CoV-2 infection were reported to have mild to severe respiratory illness with symptoms such as fever, cough and shortness of breath (Huang C. et al., 2020; Chan et al., 2020; Guan et al., 2020; Hu et al., 2020). However, there are a large special group of patients who are diagnosed by a positive RT-PCR test but are asymptomatic (Moghadas et al., 2020; Nishiura et al., 2020). It has shown that transmission via people with no symptoms could be a primary driver of COVID-19 spread (Bai et al., 2020; Moghadas et al., 2020), because the viral load in asymptomatic patients appeared to be similar to that in patients showing symptoms (Lee et al., 2020). The neglected silent spreaders have caused significant difficulties in the control of this pandemic (Moghadas et al., 2020).

Diagnosis of asymptomatic SARS-CoV-2 infection in patient is critical for controlling the spread of the disease, guiding the policies of public health, and providing therapeutic decisions. Detection assays of SARS-CoV-2 in nasal swab based on RT-PCR are the most effective method for diagnosis of COVID-19 (Bonetta, 2005). Nevertheless, the relative long detection time (typically 3–4 h) and expensive reagents compromise its advantages especially in the large-scale COVID-19 testing (Bonetta, 2005). In addition, the false negative rate of RT-PCR for COVID-19 cases is still not satisfied enough (Ai et al., 2020; Falaschi et al., 2020). Therefore, alternative reliable diagnostic techniques which could provide speedy analytical result for COVID-19 especially its asymptomatic type are quite necessary.

Matrix-assisted laser desorption/ionization mass spectrometry (MALDI-MS) have been equipped in many diagnostic laboratories around the globe (Patel, 2015). Its application to microbial identification revolutionized clinical microbiology by providing rapid identification with minimal sample preparation at a potential savings in costs. MALDI-MS enables high-throughput and ultrafast (<1 s/sample) analysis of clinical samples, and the obtained fingerprint mass spectra containing abundant information could be used to discriminate species (Patel, 2015; Wu et al., 2019; Huang L. et al., 2020). This approach is well established and accepted in the diagnostics of many important diseases. However, the applicability of MALDI-MS in the diagnosis of asymptomatic COVID-19 has not been reported. Very recently, the feasibility of MALDI-MS-based approach for the diagnosis of symptomatic COVID-19 by detecting protein components of SARX-CoV-2 in nasal swabs or peptides in serum was demonstrated (Nachtigall et al., 2020; Yan et al., 2021). We reasoned that the finer metabolic pattern of MALDI-MS in low-mass-range will be a more efficient way to identify the more challengeable asymptomatic SARS-CoV-2 infection.

Besides the direct pathogen detection, serum profiling holds promise for the early diagnosis of many diseases because of its abundant metabolic and proteomic information (Cohen et al., 2018; Huang L. et al., 2020; Shen et al., 2020; Song et al., 2020). The metabolic serum analysis, including the metabolites and lipids, is more distal over genomic and proteomic approaches for precision diagnostics (Mayers et al., 2014; Song et al., 2020). Metabolites and lipids dysregulations have recently been observed in the serum of symptomatic COVID-19 patients (Shen et al., 2020; Song et al., 2020; Delafiori et al., 2021).

In this study, we hypothesized that SARS-CoV-2 would induce characteristic metabolic alterations in the serum of asymptomatic patients that can be detected by MALDI-MS, which may contribute to the diagnosis of this special infection. To test this hypothesis, we recruited a large cohort containing 92 of asymptomatic SARS-CoV-2 infected individuals and 182 of matched healthy controls in Wuhan, China. The serum samples from the individuals were collected and tested by MALDI-MS, which provided the information of both the metabolites and lipids. We also recognized the need to adopt the machine learning methods to process MS big data to obtain necessary accuracy (Huang L. et al., 2020). By applying the developed ensemble model of machine learning to the metabolic MALDI mass spectra, asymptomatic SARS-CoV-2 infection was distinguished with a high sensitivity of 0.946 and specificity of 0.929. To the best of our knowledge, this is the first example to demonstrate the MALDI-MS combined with machine learning analysis can be used to detect asymptomatic SARS-CoV-2 infection.

## Results

### Study Design of the MALDI-MS and Machine Learning-Based Diagnosis of Asymptomatic COVID-19 Patients

**FIGURE 1:**
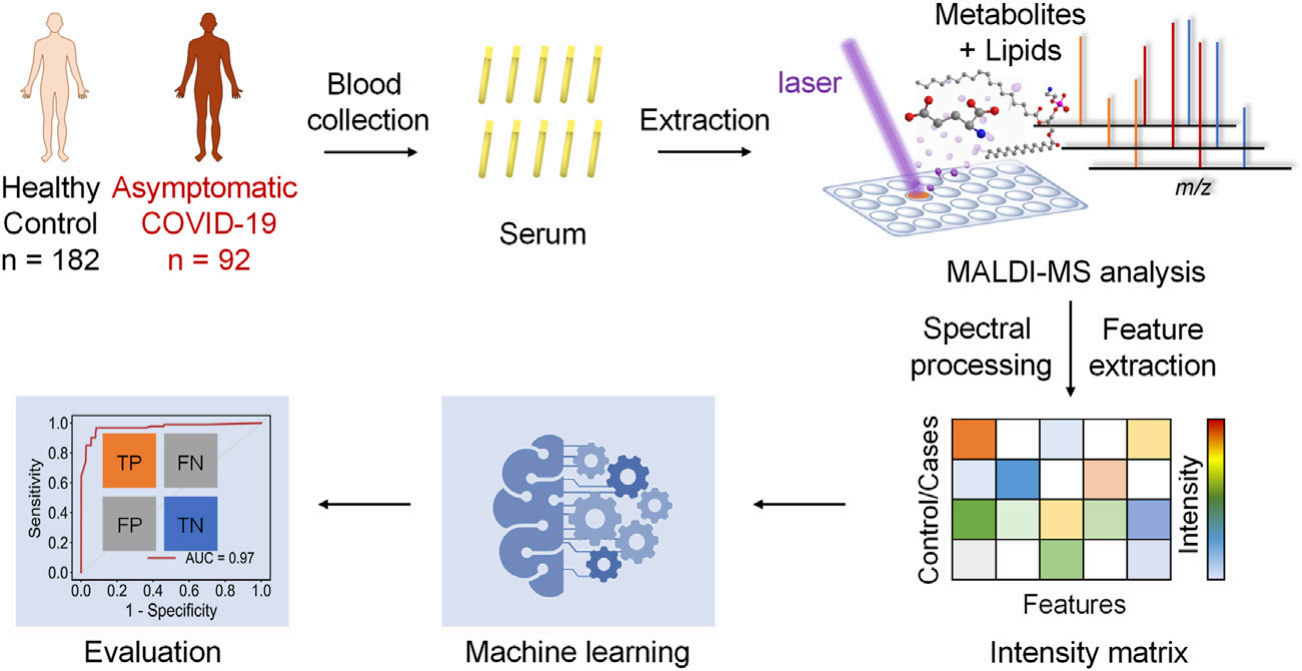
Schematics of the MALDI-MS and machine learning-based diagnosis of asymptomatic COVID-19 patients.

The hypothesis of this study is the alteration of the serum metabolic pattern caused by SARS-CoV-2 infection in asymptomatic patients could be distinguished from healthy controls by using MALDI-MS analysis and machine learning, and the extracted features would enable the accurate diagnosis (Figure 1). To identify asymptomatic individuals, the extensive screening was conducted for thousands of close contacts under quarantine in Wuhan. Individuals with positive RT-PCR results then were screened by point prevalence surveys and symptoms assessments. 92 of asymptomatic cases, defined as individuals with a positive nucleic acid test but without any relevant clinical symptoms in the preceding 14 days and during subsequent hospitalization, were included in this study (Table 1). To minimize the influence of age and gender, the stringent healthy controls (*n* = 182) were selected with exactly matched ages and similar gender ratios.

**TABLE 1:** Characteristics of sub-groups of enrolled subjects.

Variables	Groups		*p* value
	Control (n = 182)	COVID-19 (n = 92)	
Age–year			0.966
Mean ± SD	45.0 ± 13.1	45.0 ± 13.2	-
Median (IQR)	43.0 (35, 52)	42.5 (36, 53)	-
Gender, n (%)			0.996
Male	97 (53.3)	49 (53.3)	-
Female	85 (46.7)	43 (46.7)	-
IgG level			
Mean ± SD	N/A	12.8 ± 11.7	-
Median (IQR)	N/A	9.0 (3.5, 20.0)	-
IgM level			
Mean ± SD	N/A	0.7 ± 1.0	-
Median (IQR)	N/A	0.4 (0.3, 0.6)	-
RT-PCR Positive, n (%)	0 (0%)	92 (100%)	-

**FIGURE 2:**
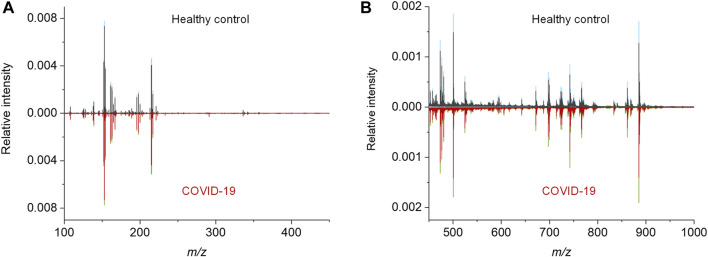
MALDI MS metabolic patterns of control and asymptomatic COVID-19 patient samples. **(A,B)** Mean MALDI mass spectra and the respective interquartile range (IQR) obtained from healthy control and asymptomatic COVID-19 groups in the mass range of **(A)** 100–450 Da and **(B)** 450–1,000 Da, respectively. The IQR were denoted by blue and green for the healthy control and patient groups, respectively.

To acquire the metabolic MALDI mass spectra of serum, the metabolites were first extracted by ethanol solution, and then subjected to MALDI-MS analysis in negative ion mode. Here, NEDC was used as the matrix, because of its relatively clean background in the low mass range and its ability to analyze metabolites and lipids simultaneously (Chen et al., 2012; Wang et al., 2015). Fifty of quality control (QC) samples prepared by pooled serum extracts were added in between cohort samples to examine the reproducibility of this MALDI MS method and check the experimental stability during the MS acquisition. The m/z features with S/N > 3 were extracted and 238 of common features were obtained in these samples. To compensate the signal variability among samples, the intensity of each m/z feature was normalized to the total ion current of each mass spectrum. We plotted the heat map of all the 50 independent metabolic patterns from QC samples, showing that the feature signals were distributed vertically and uniformly (Supplementary Figure S1A). In addition, the relative standard deviation (RSD) of these features were calculated using their normalized intensities in 50 QC samples, and over 87% (208/238) of m/z features show RSD less than 30% (Figure S1b). These results demonstrate the good reproducibility of this MALDI MS method, and also indicate the validity of the normalization strategy. Subsequently, the MALDI mass spectra of all the 274 cohort samples were preprocessed (Figure 2). The peaks were also extracted with S/N > 3, and only those which were present in more than 80% spectra of all samples were retained. The final intensity matrix was obtained with 219 features (Supplementary Datasheet 1), by estimating the threshold and deciding the background noise on the maximum interclass variance and excluding random background peaks according to the threshold, including the species located in the mass ranges of metabolites (Figure 2A) and lipids (Figure 2B). These 219 of m/z features were considered as the preliminary MS output (metabolic pattern) for the COVID-19 classifier. The heat map of all the 274 independent metabolic patterns from control and asymptomatic COVID-19 patients shows that the metabolite signals were distributed uniformly in the given m/z range (Supplementary Figure S1). This result indicates the reliability of the serum metabolic patterns obtained with NEDC-assisted laser desorption/ionization mass spectrometry (LDI MS).

### Diagnosis of Asymptomatic COVID-19 by Machine Learning

To evaluate the possibility for diagnosis with MALDI MS-based serum metabolic patterns, we examined different algorithms for the discrimination of asymptomatic COVID-19 from healthy controls. First, we used unsupervised learning methods principal component analysis (PCA) and uniform manifold approximation and projection (UMAP) to reduce dimensions of the intensity matrix and compare the two groups of samples in a multidimensional space using all 219 peaks. As shown in (Supplementary Figures 2A,B), the control and patient samples could not be well separated, which may imply the subtle differences between asymptomatic and healthy groups. Therefore, more advanced methods are required to discriminate them.

We then try to apply five different machine learning algorithms to classify the control (n = 182) and asymptomatic COVID-19 (n = 92) samples: SVM, KNN, RF, MLP, and XGB. Fivefold (outer) nested repeated (ten times) tenfold (inner) cross-validation was used for hyperparameters optimization and performance evaluation (Supplementary Table S1) (Krstajic et al., 2014). The hyperparameters of each model were optimized through repeated tenfold cross-validation in inner loop. The performance of each model was comprehensively evaluated by several indicators calculated in outer loop, namely receiver operating characteristic (ROC) curve, precision-recall (PR) curve, accuracy, sensitivity, and specificity. The comparison between true positive rate (TPR) and false positive rate (FPR) at various thresholds were performed through ROC curve (Supplementary Figure S2C) and the comparison between precision and recall were measured through PR curve (Supplementary Figure S2D). Accuracy, sensitivity, and specificity respectively measures the proportion of all samples, positive samples and negative samples that were correctly predicted (Supplementary Figure S2E and Table S2) using all the 219 features. The results of the machine learning models show significant improvement over PCA and UMAP, however, the accuracies (≤0.891) and sensitivities (≤0.740) still need to be improved.

**FIGURE 3:**
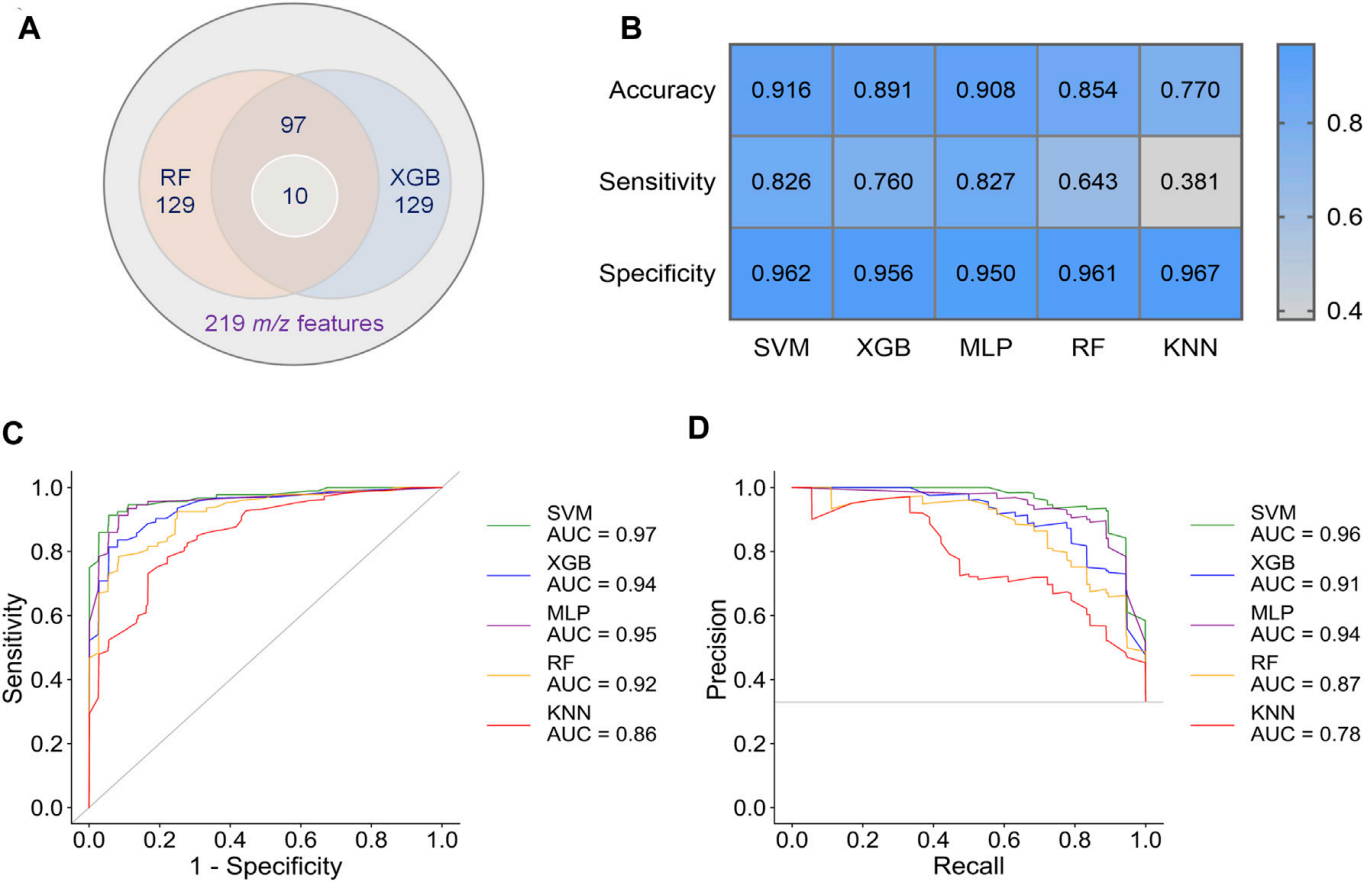
Results of preliminary classification and machine learning analysis with 97 features. **(A)** Venn diagram of the feature selection. 97 common features with high importance in both RF and XGB models were selected from 219 m/z features. Then, 10 of biomarkers were found from these 97 features. **(B)** Performance indicators of five separate machine learning models. **(C)** ROC curves of five separate machine learning models. **(D)** PR curves of five separate machine learning models.

To obtain better classification, we did a feature selection before the machine learning. Model-based ranking was used due to its compatibility with MALDI data compare with information gain and correlation-based methods (Nachtigall et al., 2020). A new intensity matrix was generated containing 97 features (Supplementary Datasheet 2), which importance ranked high in both RF and XGB models (Figure 3A), and was put into classifiers (Supplementary Table S3). The models with selected features show better performance in accuracy, sensitivity, and specificity than that with unselected features (Figures 3B–D and Supplementary Table S4).

By comparing the metrics among five machine learning models, we found that SVM, XGB and MLP achieved higher performance than RF and KNN (Figures 3C–E). The area under curve (AUC) could reach to 0.97, 0.94, and 0.95 for the models SVM, XGB and MLP, respectively (Figure 3C). Accordingly, the accuracy rate could reach 91.6% in the SVM model. For the models SVM, XGB, and MLP, we also noted that the specificities of are very high (0.950–0.962), whereas the sensitivities are relatively low (0.760–0.827). Given the much high infectivity of COVID-19, it is critical to discriminate the virus-carrying asymptomatic patients in the first test. Therefore, sensitivity is a very important indicator of our classifiers in this MALDI-MS-based pre-diagnosis of COVID-19.

**FIGURE 4:**
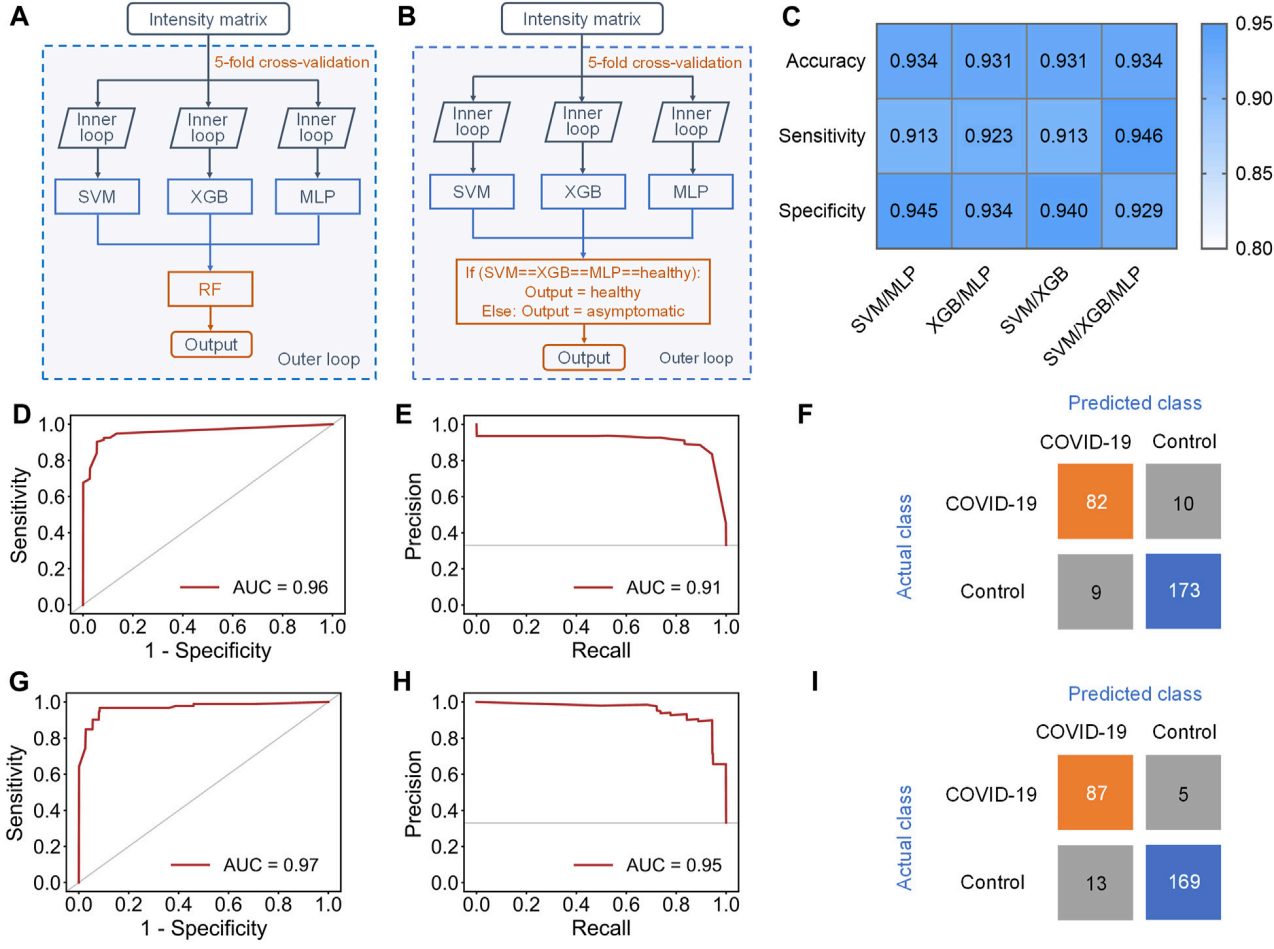
Results of stacking and ensemble learning methods. **(A)** Schematic workflow of the stacking model with five-fold nested cross-validation, including the inner loop to tune the optimized hyperparameters of each separate classifier and the outer loop to evaluate the performance of the stacking model. **(B)** Schematic workflow of the new ensemble model with five-fold nested cross-validation, including the inner loop to tune the optimized hyperparameters of each separate classifier and the outer loop to evaluate the performance of this ensemble model. **(C)** Performance indicators of four new ensemble models with novel voting algorithm. **(D,E)**, **(D)** ROC and **(E)** PR curves of the stacking model containing SVM, XGB and MLP in the first level. **(F)** Confusion matrix of the stacking model. **(G,H)**, **(G)** ROC and **(H)** PR curve of the new ensemble model containing SVM, XGB and MLP in the first level. **(I)** Confusion matrix of the ensemble model.

To address this issue, an ensemble learning scheme named stacking (Li et al., 2019) was tried to combine multiple machine learning models, aiming at improving sensitivity while maintaining accuracy (Figure 4A). Stacking is a general two-level framework that uses a learning algorithm as a specific combination method (Li et al., 2019). The first level is consisted of multiple machine learning models. In the second level, a meta-learner takes the output of classifiers in first level as input to generate the final output of whole model. Due to the lower performance of RF and KNN in this case, at least two of SVM, XGB and MLP were randomly selected to form the first layer of the stacking model. When the first level contained SVM, XGB, and MLP, the stacking model with RF as the meta-learner could reach 0.931 ± 0.033 accuracy and 0.891 ± 0.069 sensitivity (Supplementary Table S5). The AUC of ROC curve and AUC of PR curve of the stacking model could achieve 0.96 and 0.91, respectively (Figures 4D,E), and 82/92 asymptomatic COVID-19 samples and 173/182 healthy controls were correctly diagnosed (Figure 4F). Although the accuracy and sensitivity of the stacking model were slightly improved compared to individual models, the sensitivity was still relatively lower than the accuracy and specificity.

Therefore, we finally proposed a novel voting algorithm to replace the meta-learner in the second level, making the model focus more on the classification of asymptomatic samples (Figure 4B). In the voting algorithm, a sample will be predicted as healthy only when the outputs of all classifiers in first level are healthy. This model exhibited highest performance when SVM, XGB, and MLP were contained in the first level (Figure 4C and Supplementary Table S6). The ensemble model with the new voting algorithm reached accuracy of 0.934 ± 0.029 and sensitivity of 0.946 ± 0.033. The AUC of ROC curve and PR curve could achieve 0.97 and 0.95 (Figures 4G,H). Obviously, the overall performance of the ensemble model with new voting algorithm was not only much better than that of separate models, but also better than that of the stacking models. Based on this algorithm, 87/92 asymptomatic COVID-19 samples and 169/182 healthy controls were correctly diagnosed (Figure 4I).

### Construction of the Metabolic Biomarker Panel

**FIGURE 5:**
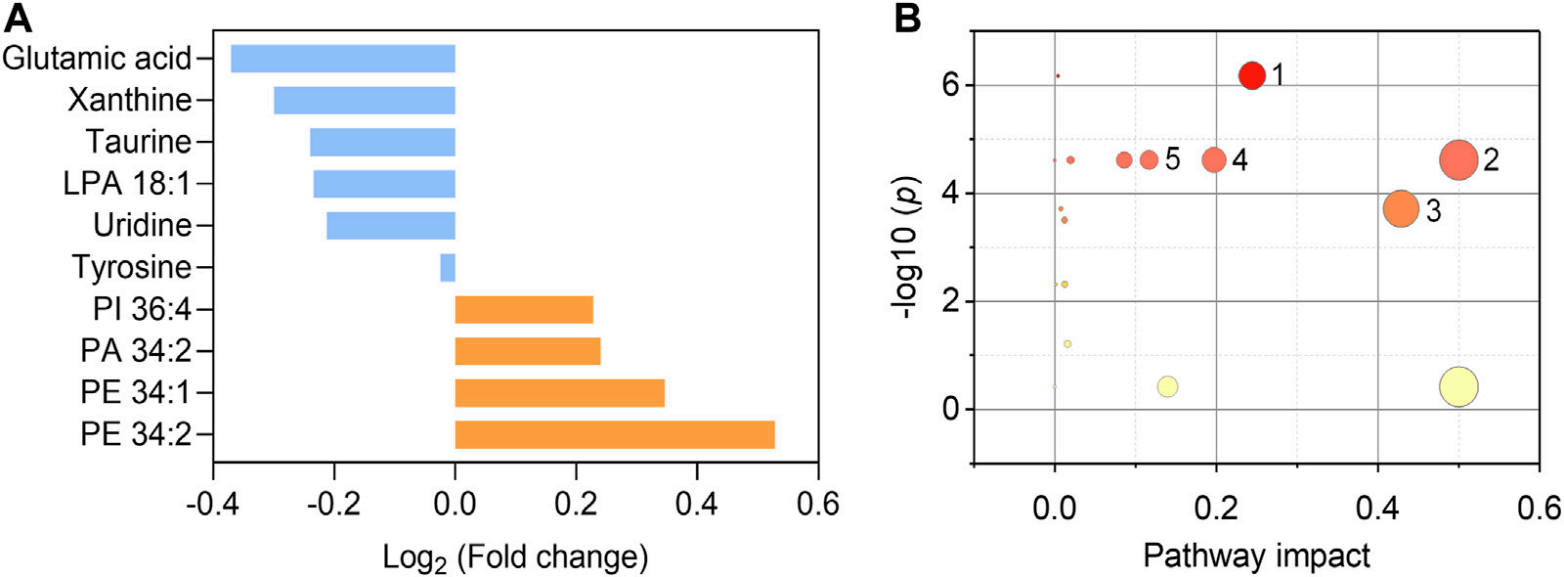
Fold changes of the ten biomarkers and potential pathways alteration. **(A)** Fold changes of six down-regulated metabolites (blue) and four up-regulated phospholipids (orange) in asymptomatic COVID-19 patients compared with healthy controls. **(B)** Potential pathways differentially regulated in asymptomatic COVID-19 patients vs. healthy controls. The ten selected metabolites were tested to identify altered pathways. The color and size of each circle were correlated to the *p* values and pathway impact values, respectively. A total of five pathways were considered as altered (*p* < 0.05, pathway impact >0.1): (Zhou et al., 2020) Glycerophospholipid metabolism; (Wu et al., 2020) Glutamine and glutamate metabolism; (Huang C. et al., 2020) Taurine and hypotaurine metabolism; (Guan et al., 2020) Alanine, aspartate, and glutamate metabolism; (Chan et al., 2020) Arginine biosynthesis.

We further endeavor to find metabolic biomarkers in patterns to characterize relevant pathways and potential therapeutic targets. We confirmed a biomarker panel containing ten metabolites and lipids based on the performance to distinguish the asymptomatic COVID-19 from controls with machine learning models (Figure 5A, Supplementary Tables S7–9). The structural identification of these compounds was based on accurate mass measurement and tandem MS (Supplementary Figures S3, 4). This panel consists of phospholipids and amino acids, purine, and nucleoside, including PE 34:1 (16:0/18:1), PE 34:2 (16:0/18:2), PI 36:4 (16:0/20:4), PA 34:2 (16:0/18:2), LPA 18:1, glutamic acid, tyrosine, taurine, xanthine, and uridine (Figure 5A and Table S7). Notably, we found that glutamic acid was the most down-regulated (*p* = 7.66E-6) species, while PE 34:2 was the most up-regulated (*p* = 4.28E-6) species (Supplementary Figures 5, 6). Three individual machine learning models including SVM, XGB, and MLP, and the proposed ensemble machine learning model, were applied to classify all the 274 control and asymptomatic COVID-19 samples with the ten biomarkers. For each model, fivefold (outer) nested repeated (ten times) tenfold (inner) cross-validation (with randomized stratified splitting) was used for hyperparameters optimization and performance evaluation (Supplementary Table S8). The results showed that one single model cannot be very efficient in discriminating COVID-19 from controls (AUC ≤0.834, Sensitivity ≤0.642, Supplementary Table S9). However, the ensemble model containing SVM, XGB and MLP in the first level accounted for an enhanced AUC of 0.850 with the sensitivity of 0.837, which indicated the ability to distinguish asymptomatic COVID-19 from noninfected ones. The construction of the biomarker panel could simplify the analysis and facilitate the large-scale clinical use of this approach.

To interrogate the potential metabolic pathway alteration contributed by these metabolites, pathway analysis (Figure 5B) was conducted in MetaboAnalyst (https://www.metaboanalyst.ca/). A total of five pathways were considered as altered (*p* < 0.05, pathway impact value >0.1): (Zhou et al., 2020) Glutamine and glutamate metabolism; (Wu et al., 2020) Glycerophospholipid metabolism (Supplementary Figure S7); (Huang C. et al., 2020) Taurine and hypotaurine metabolism; (Guan et al., 2020) Alanine, aspartate, and glutamate metabolism; (Chan et al., 2020) Arginine biosynthesis. These results are consistent with the known fact that viral infection rewires host cell metabolism to facilitate optimal viral replication (Thaker et al., 2019; Xiao et al., 2020). The significant decrease of the glutamic acid in the serum of asymptomatic COVID-19 patients indicated the dysregulation of glutamine and glutamate metabolism during the viral infection cycle (Bharadwaj et al., 2020), as well as the alteration of taurine and hypotaurine metabolism implied by the decrease of the concentration of taurine (Figure 5A and Supplementary Figure S6). These findings are consistent with the recent studies that the suppression of amino acid metabolism was observed in COVID-19 patients (Shen et al., 2020; Bruzzone et al., 2020; Thomas et al., 2020), which might be related to the dysregulation of hepatic metabolism (Shen et al., 2020; Bruzzone et al., 2020). Besides, the metabolic pathway of glycerophospholipid was also significantly influenced in asymptomatic COVID-19 (Figure 5B). Viruses are known to induce profound changes in host cell lipidomes and usurp key energy pathways in their exploitation of host metabolic resources for fueling the different stages of viral infection (Kyle et al., 2019; Bruzzone et al., 2020; Song et al., 2020). We found the increase of glycerophospholipids including PE 34:1, PE 34:2, PI 36:4, and PA 34:2 in asymptomatic COVID-19, whereas the lysophospholipid LPA 18:1 was reduced. The increase of PE and PI was reported in symptomatic COVID-19 (Song et al., 2020), and corroborated a previous study on plasma lipid alterations in Ebola virus disease survivors compared to healthy controls (Kyle et al., 2019). Phospholipids are the major components of plasma membrane and circulating lipoproteins (Dashti et al., 2011). Increases of these lipids were possibly reflecting the augmented secretion of them into the circulation (Song et al., 2020). Notably, the opposite changes of increased PA and decreased LPA might suggest the disruption of the balance between them. This distinct metabolic and lipid dysregulation of asymptomatic COVID-19 provided new insight for understanding its unique mechanism.

## Discussion

The asymptomatic COVID-19 patients are silent spreaders who make it more difficult for the prevention and control of the epidemic. Thus, the rapid identification of asymptomatic COVID-19 is an urgent need. Current diagnosis of asymptomatic COVID-19 mainly depends on RT-PCR, which is time-consuming and compromised by the moderate sensitivity (Bonetta, 2005; Ai et al., 2020; Falaschi et al., 2020). In addition, the lack of clinical oversight of asymptomatic COVID-19 make the recruitment of large clinical cohort quite difficult. So far, we still know very little about the asymptomatic COVID-19 infection. In this study, we compared the metabolic profile between the healthy control and asymptomatic COVID-19, and found the MALDI MS-based serum metabolic profiling combined with machine learning could be an alternative method to discriminate the infected patients from controls with high accuracy. These preliminary results indicated the great potential of this new approach for the development of a meaningful diagnostic method. Although RT-PCR are undoubtedly useful for COVID-19 diagnosis, this MALDI-MS-based serum metabolic approach was validated as a promising alternative given its speed, simplicity, high-throughput, and the availability of equipment and expertise in many core facilities in developing countries. The serum samples were simply extracted with common solvents, and no other expensive reagent was needed for MALDI MS analysis. The high-throughput enabled the analysis of 384 samples in one MALDI target plate within 10 min (∼seconds/sample). So, the average cost for one sample would be lower than RT-PCR especially in the large-scale analysis.

This study may also have some limitations. Firstly, while gender and age were matched between asymptomatic patients and healthy controls in this cohort, the information of BMI was not included due to the constraints in collecting these medical records during the outbreak, which might be potential confounders in this study. In addition, restricted by the controlled healthcare resource during the pandemic, collection of symptomatic COVID-19 patients and multicenter cohorts with stringently matched demographics were not available. However, to minimize the overfitting of our machine learning models, fivefold (outer) nested repeated (ten times) tenfold (inner) cross-validation (with randomized stratified splitting) was used for each model, for hyperparameters optimization and performance evaluation.

## Conclusions

This work validated the hypothesis that the rapid diagnosis of asymptomatic COVID-19 could be achieved by serum metabolic analysis combined with developed machine learning method. The distinct metabolic pattern with a panel of biomarkers may provide clues for deep understanding the unique mechanism of asymptomatic SARS-CoV-2 infection. This approach would play an important role in the large-scale screening assay of SARS-CoV-2 in regions that lack of adequate resource of RT-PCR assay.

## Data Availability

The original contributions presented in the study are included in the article/Supplementary Material, further inquiries can be directed to the corresponding author.

## Abbreviations

RT-PCR, reverse transcription polymerase chain reaction; RT-qPCR, reverse transcription quantitative polymerase chain reaction; MALDI-MS, matrix-assisted laser desorption/ionization mass spectrometry; LDI MS, laser desorption/ionization mass spectrometry; COVID-19, coronavirus disease 2019; SARS-CoV-2, severe acute respiratory syndrome coronavirus 2; TIC, total ion current; PCA, Principal Component Analysis; UMAP, Uniform Manifold Approximation and Projection; SVM, Support Vector Machine; KNN, K-Nearest Neighbor; RF, Random Forest; MLP, Multi-Layer Perceptron; XGB, XGBoost; AUC, average under curve; ROC curve, receiver operating characteristic curve; PR curve, precision-recall curve; TPR, true positive rate; FPR, false positive rate; NEDC, N-(1-naphthyl) ethylenediamine dihydrochloride; PCA, principal component analysis.
