# Supplementary material for: Machine Learning of Serum Metabolic Patterns Encodes Asymptomatic SARS-CoV-2 Infection
Source: Front Chem. 2021 Oct 1;9:746134. doi: 10.3389/fchem.2021.746134 (PMC8517325; doi:10.3389/fchem.2021.746134)
Supplement: Supplementary file 2 [file DataSheet1.pdf]

Supplementary Information

Machine Learning of Serum Metabolic Patterns Encodes Asymptomatic SARS-CoV-2 Infection

Qiongqiong Wan,[1,5] Moran Chen,[1,5] Zheng Zhang,[2,5] Yu Yuan,[3,4,5] Hao Wang,[3,4] Yanhong Hao,[1] Wenjing Nie,[1] Liang Wu,[1] Suming Chen*[1]

[1]Institute for Advanced Studies, Wuhan University, Wuhan, Hubei 430072, China

[2]Central China Normal University, Wuhan, Hubei 430079, China

[3]Huazhong University of Science & Technology, Department of Occupational and Environmental Health, Hubei Key Laboratory of Environmental Health (Incubating), Wuhan, Hubei 430030, China.

[4]Huazhong University of Science & Technology, Wuhan, Hubei 430030, China.

[5]These authors contributed equally

*Correspondence: sm.chen@whu.edu.cn (S.C.)

Materials

N-(1-naphthyl) ethylenediamine dihydrochloride (NEDC), L-Glutamic acid, Xanthine, Taurine, Uridine, L-Tyrosine and Glucose were purchased from J&K Scientific LTD, LPE 18:1(9*Z*), PE 18:1(9*Z*) / 18:1(9*Z*), Cer 18:1/16:0, PS 16:0/18:1(9*Z*) (sodium salt), PG 18:0/18:1(9*Z*) (sodium salt) were purchased from Sigma-Aldrich. Ultrapure water (over 18 MΩ·cm) from a Milli-Q reference system (Millipore) was used throughout. Other solvents and reagents used were of HPLC analytical grade. $[Cl]^-$, $[HCl+Cl]^-$ and $[Glucose+Cl]^-$ were used to calibrate MALDI TOF mass method between 0-450 Da. LPE 18:1(9*Z*), PE 18:1(9*Z*) / 18:1(9Z), Cer 18:1/16:0, PS 16:0 / 18:1(9*Z*) (sodium salt), PG 18:0 / 18:1(9*Z*) (sodium salt) were used as calibration mixture to calibrate MALDI TOF mass method between 450-1000 Da.

Methods

Cohort recruitment and data collection

We retrospectively recruited a total of 92 asymptomatic COVID-19 patients who were under quarantine in Wuhan from January to March, 2020. They were diagnosed as asymptomatic COVID-19 according to the Chinese Government Diagnosis and Treatment Guideline (Trial 6th version) (NHCPRC, 2020). Briefly, asymptomatic COVID-19 referred to those people who meet the following two clinical criteria: 1) exhibited no typical clinical symptoms, 2) tested either positive for SARS-CoV-2 nucleic acid test in respiratory specimens or seropositive for IgM antibody test. All enrolled patients were confirmed positive for SARS-CoV-2 nucleic acid except for three individuals whose RT-PCR test turned from positive to negative at the blood collection time, they were also classified as asymptomatic due to the seropositive of IgM or IgG. According to the epidemiologic investigations, none of these patients developed symptoms until they were tested negative for SARS-CoV-2. For each confirmed patient, two healthy people of the same gender and approximate age was matched as controls. These

healthy controls come from the physical examination population in Wuhan Prevention and Treatment Center for Occupational Diseases during the sample collection period.

For identification of SARS-CoV-2 infections, throat swabs were collected and tested by real-time polymerase chain reaction (RT-PCR) using virus nucleotide acid extraction kit (Shanghai Zhijiang, China, NO. P20200201) and detection kit (triple fluorescence PCR, Shanghai Zhijiang, China, NO. P20200203) according to manufacturer instructions. Briefly, target genes including RdRp, E and N were simultaneously amplified and tested during RT-PCR. Patients were diagnosed as positive if RdRp gene was positive (Ct < 43), and one of E or N was positive (Ct < 43). Patients were also diagnosed as positive if two sequential tests of RdRp were positive while E and N were negative. IgG and IgM against SARS-CoV-2 were detected in serum samples using chemiluminescence immunoassay kits (Orienter Biotechnology Co., Ltd, Sichuan, China) and Access2 automatic microparticle chemiluminescence immunoassay system (BechmanCoulter, California, USA). Antibody levels were expressed as the ratio of the chemiluminescence signal over the cutoff value (S/CO). The result was defined as positive if the S/CO value is higher than 1.00.

This study was approved by the Ethics Review Commission of WuHan Prevention and Treatment Center for Occupational Diseases (reference no. 202002).

Extraction of metabolites

Peripheral blood samples for all participants were collected using serum separation tubes after an overnight fast. Serum was separated by centrifugation at 1,500 g for 10 min and then stored at − 80 ^{o}C after standard diagnostic tests. Before used, the frozen serum was slowly thawed at 4 ^{o}C overnight. For virus inactivation and metabolites extraction, pre-chilled ethanol was added to each sample to make a final solution of 75% (v/v) ethanol. The mixture was shaken vigorously for 5 min to ensure inactivation of virus. The supernatant was collected by centrifuged at 1,2000 g for 15 min. A pooled sample was generated by taking equal aliquot of each experimental sample to serve as a technical replicate which was run multiple times throughout the experiment. Extracted water samples served as blanks. All samples were stored at − 80 ^{o}C until analysis.

MALDI-MS analysis

Mass spectrometric analyses were performed with a MALDI time-of-flight instrument (Autoflex Max, Bruker) with a pulsed Nd: YAG laser (355 nm), operating in negative-ion reflection mode. The high voltages for ion source 1 and 2 are 19.1 kV and 16.8 kV, respectively. The high voltages for reflector and reflector 2 are 21.1 kV and 9.7 kV, respectively. The matrix solution was prepared with *N*-(1-naphthyl) ethylenediamine dihydrochloride (NEDC, 10 mg/mL) at 30:70 (v/v) ethanol and deionized water. One microliter of each extracted serum with mixed with 1 μL of matrix, and 1 μL of mixture was spotted on a MALDI steel plate and air-drying. Spectra were auto-generated by summing 2000 single spectra (5 × 400 shots) with the laser frequency of 2000 Hz in the range between 0-450 Da or 450-1000 Da by shooting the laser at random positions on the target spot.

Quality control (QC) samples were prepared by pooling the extracts of each sample. Four QC samples were analyzed at the start of batch and then one QC sample was analyzed at every six cohort samples. In total, 50 of QC samples were analyzed with the same MALDI MS conditions as the cohort samples, and the mass spectra was processed with the same procedures.

Spectral preprocessing

MALDI mass spectra were processed with Flex analysis software, and the peak list with signal-to-noise more than 3 were exported for further analysis. The intensity was normalized using the total ion current (TIC) calibration method. The peaks of each sample were extracted to '.xlsx' files, and then converted to '.CSV' files using Excel. The following processes are implemented by R software. The '.txt' raw files were used to calculate the TIC of each sample. Then, the intensity of each extracted peak in '.CSV' file divided the corresponding TIC to generate the relative intensity. Peaks were binned with the tolerance of 0.05. And only peaks which were present in more than 80% spectra of all samples would be retained. The blank value of each m/z were replaced by the one-tenth of the minimum non-zero value in all samples. Through the above spectral preprocessing, there were 184 features in m/z range 0-450 and 68 features extracted in m/z range 450-1000. Then we integrated all features together and delete the features which had m/z less than 100 to generate the final intensity matrix

with 219 features. The intensity matrix would be used for statistical analysis, Principal Component Analysis (PCA), Uniform Manifold Approximation and Projection (UMAP) and machine learning.

Statistical analysis

Two-tailed Wilcoxon rank sum test with BH corrections was used to measure whether each feature had a significant difference between asymptomatic and healthy. If the adjusted P value < 0.05, the feature would be considered to have significant difference between two kinds of samples. The statistical analysis was performed using R (version 4.0.2).

PCA

PCA is a linear unsupervised machine learning algorithm that can be used for data dimension reduction. PCA was performed on all samples using Python package scikit-learn (n_components = 0.9) (version 0.22.1) and matplotlib (version 3.1.3).

UMAP

UMAP is a nonlinear dimension reduction technique that can be used for data visualization. UMAP was performed on all samples using Python package umap-learn (version 0.4.4) with parameters (n_neighbors = 14, min_dist = 0.5, n_components = 5, random_state = 44).

Machine learning

Five machine learning algorithms were separately used to classify the asymptomatic and control samples, including Support Vector Machine (SVM), K-Nearest Neighbor (KNN), Random Forest (RF), Multi-Layer Perceptron (MLP) and XGBoost (XGB). Before imported to model, the intensity matrix was preprocessed using different methods.

Combined zero-mean normalization and PCA (n_components = 0.99) were used for SVM and KNN. Only zero-mean normalization was used for MLP, RF and XGB used the intensity matrix without preprocessing.

Feature selection was carried out through model-based ranking. We put the intensity matrix with 219 features into RF and XGB respectively. Then calculate the mean value of feature importance obtained from ten training, and select 97 features ranking top 130 in both RF and XGB.

For each model, fivefold (outer) nested repeated (ten times) tenfold (inner) cross-validation (with randomized stratified splitting) was used for hyperparameters optimization and performance evaluation. The hyperparameters of each algorithm were optimized in the inner loop by grid search. These hyperparameters included C and gamma for SVM, n_neighbors and weights for KNN, n_estimators and max_depth for RF, the number of nodes for each layer for MLP (three layers), max_depth and learning_rate for XGB. Then through repeated tenfold cross-validation in the inner loop, the models obtained with the best results were reported to the outer loop, according to the average under curve (AUC) of the receiver operating characteristic (ROC) curve. The performance of each classifier was comprehensively evaluated by several indicators in outer loop, namely accuracy, sensitivity, specificity, AUC of ROC curve and AUC of precision-recall (PR) curve.

Considering the low sensitivity of each separate classifier, we further tried stacking scheme to combine different machine learning algorithms. At least two of SVM, XGB and MLP were randomly selected to form the first level of the stacking ensemble model. RF was used as the meta-learner in the second level. Due to the relatively low sensitivity of stacking model, the ensemble model with a novel voting algorithm which replaces the meta-learner in the second level was proposed. The ensemble model with the new voting algorithm, containing SVM, XGB and MLP in the first level, was found to have the best comprehensive performance. The new voting algorithm can be described as follows:

If (svm == xgb == mlp == healthy):   output = healthy   else: output = asymptomatic

In above algorithm, SVM, XGB and MLP were the predictions of the same sample obtained by the three classifiers respectively, and output was the prediction of the ensemble model. For the new model, nested cross-validation was also used for hyperparameters optimization and performance estimation. Hyperparameters optimization was carried out as same as the separate machine learning

model. In the outer loop, the new model prediction algorithm was used for calculating the indicators described above to comprehensively evaluate performance of models. Except for XGB, all machine learning algorithms, zero-mean normalization and PCA were carried out in Python (version 3.7.7) with the package scikit-learn (version 0.22.1). The Python package XGBoost (version 0.90) was used for building XGB model. ROC curve and PR curve were generated by the package matplotlib (version 3.1.3).

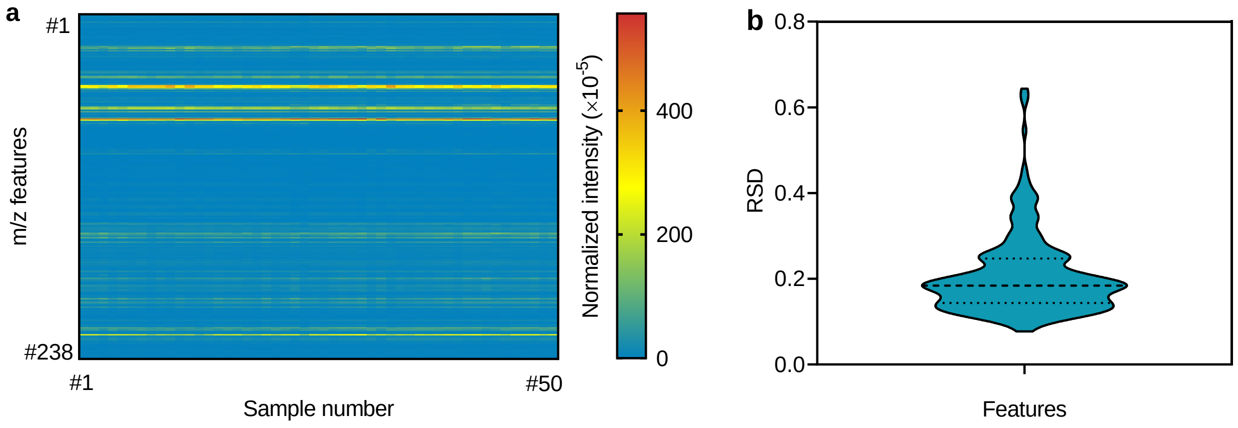

Figure S1. Characterization of the analytical results for quality control samples. a, Heat map of 50 QC patterns based on 238 of m/z features (S/N > 3). The intensity of each feature was normalized by the total ion current of individual mass spectrum. **b**, Relative standard deviation (RSD) of the 238 m/z features calculated using their normalized intensities in 50 QC samples. 208 (87.4%) of m/z features show RSD < 30%.

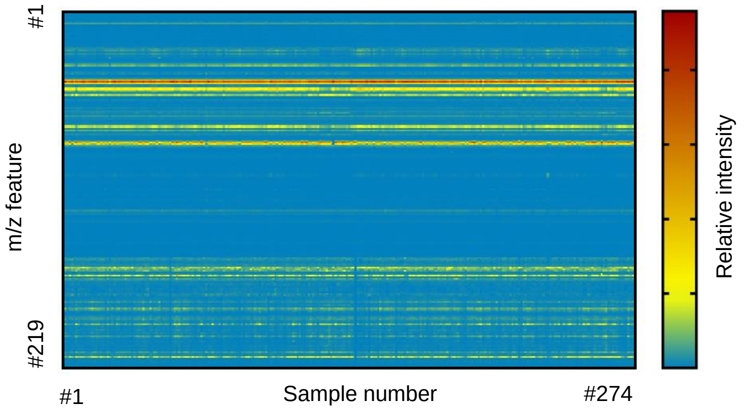

Figure S2. Heat map of 274 independent metabolic patterns based on 219 of m/z features.

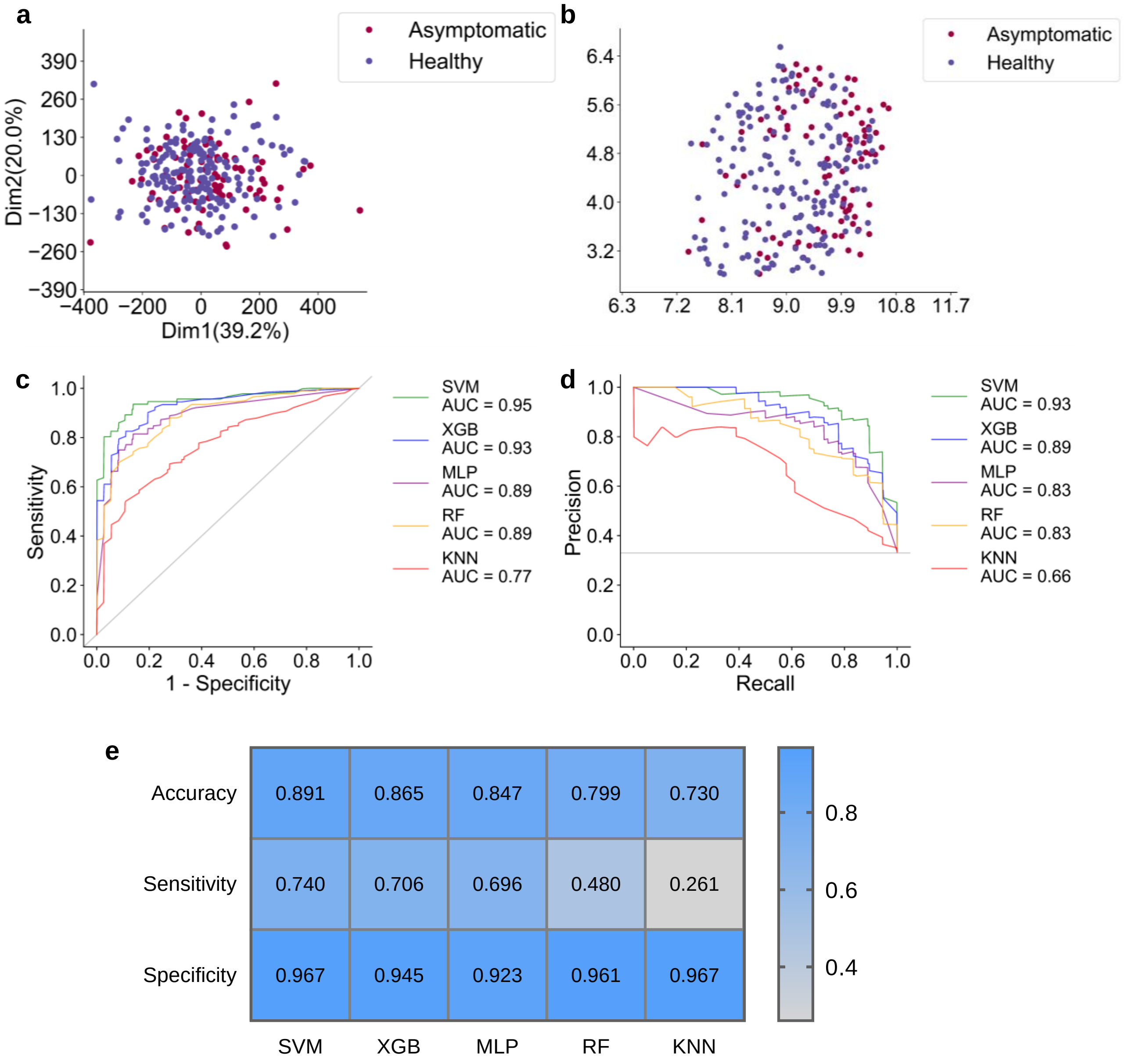

Figure S3. Results of preliminary classification and machine learning analysis using 219 features. a, PCA of asymptomatic and healthy samples using intensity matrix. **b**, UMAP analysis of asymptomatic and healthy samples using intensity matrix. **c**, ROC curves of five separate machine learning models. **d**, PR curves of five separate machine learning models. **e**, Performance indicators of five separate machine learning models.

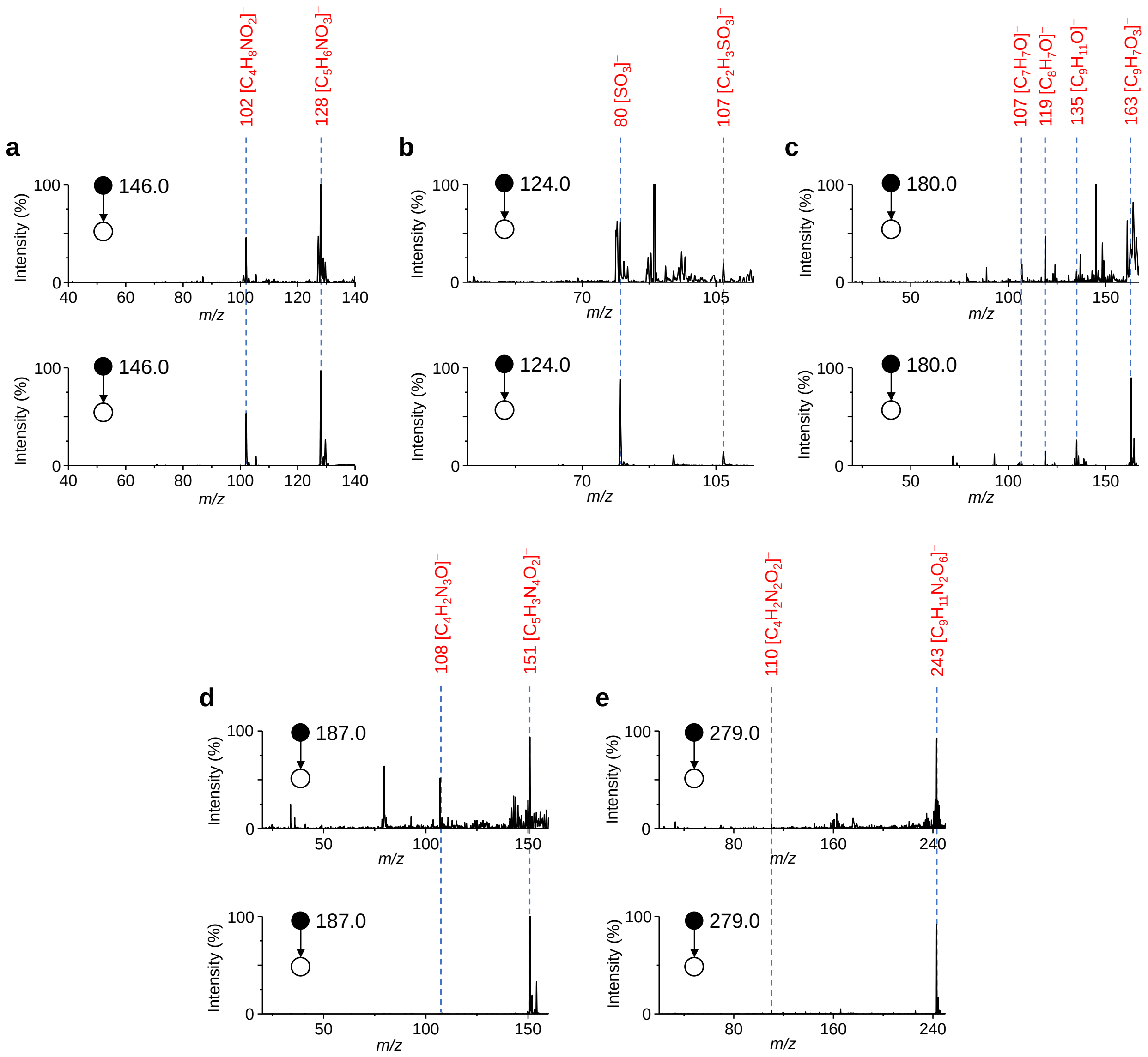

Figure S4. MALDI MS/MS spectra of glutamic acid, taurine, tyrosine, xanthine, and uridine. a-e, MS/MS spectra of $[M-H]^-$ at **a**) m/z 146.0 (glutamic acid), **b**) m/z 124.0 (taurine), **c**) m/z 180.0 (tyrosine), and $[M+Cl]^-$ at **d**) m/z 187.0 (xanthine), **e**) m/z 279.0 (uridine). The top plots in each panel denote the MS/MS spectra conducted with serum sample, and the bottom plots in each panel denote the MS/MS spectra of corresponding standards. The aligned peaks in each panel denote the matched fragments between the samples and standards.

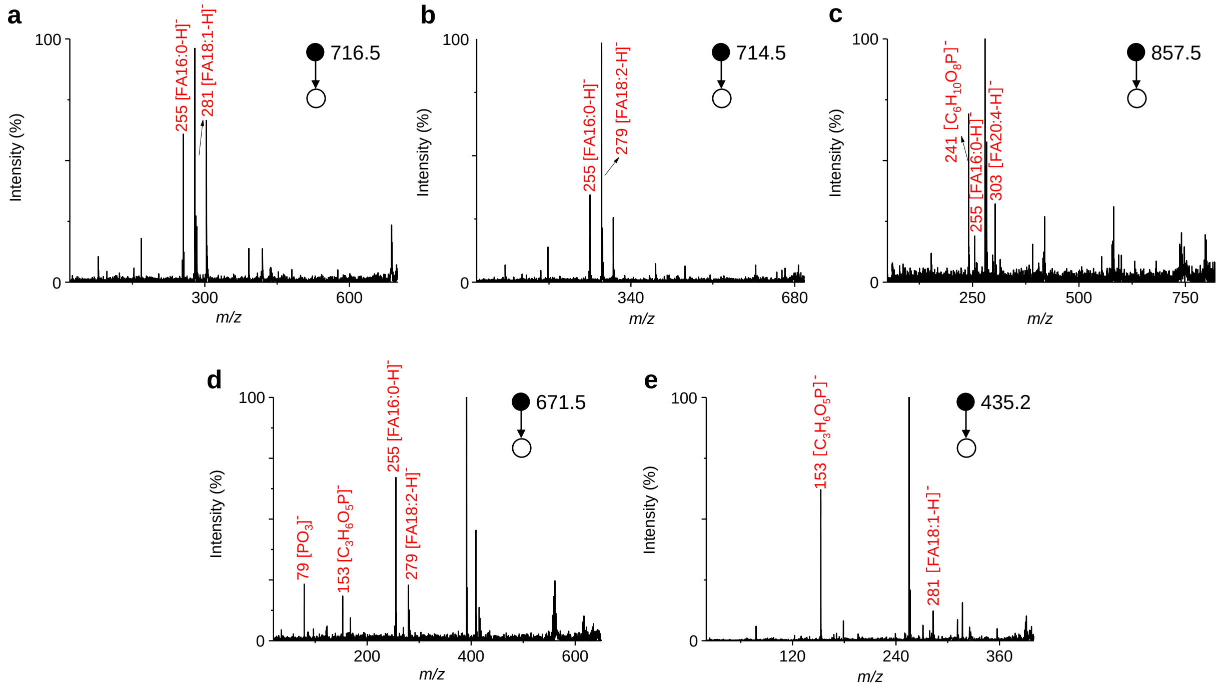

Figure S5. MALDI MS/MS spectra of phospholipids from serum sample. a-e, MS/MS spectra of $[M{-}H]^-$ at **a**) m/z 716.5 (PE 34:1 (16:0/18:1)), **b**) m/z 714.5 (PE 34:2 (16:0/18:2)), **c**) m/z 857.5 (PI 36:4 (16:0/20:4)), **d**) m/z 671.5 (PA 34:2 (16:0/18:2)), and **e**) m/z 435.2 (LPA 18:1). The aligned peaks in each panel denote the matched fragments between the samples and standards.

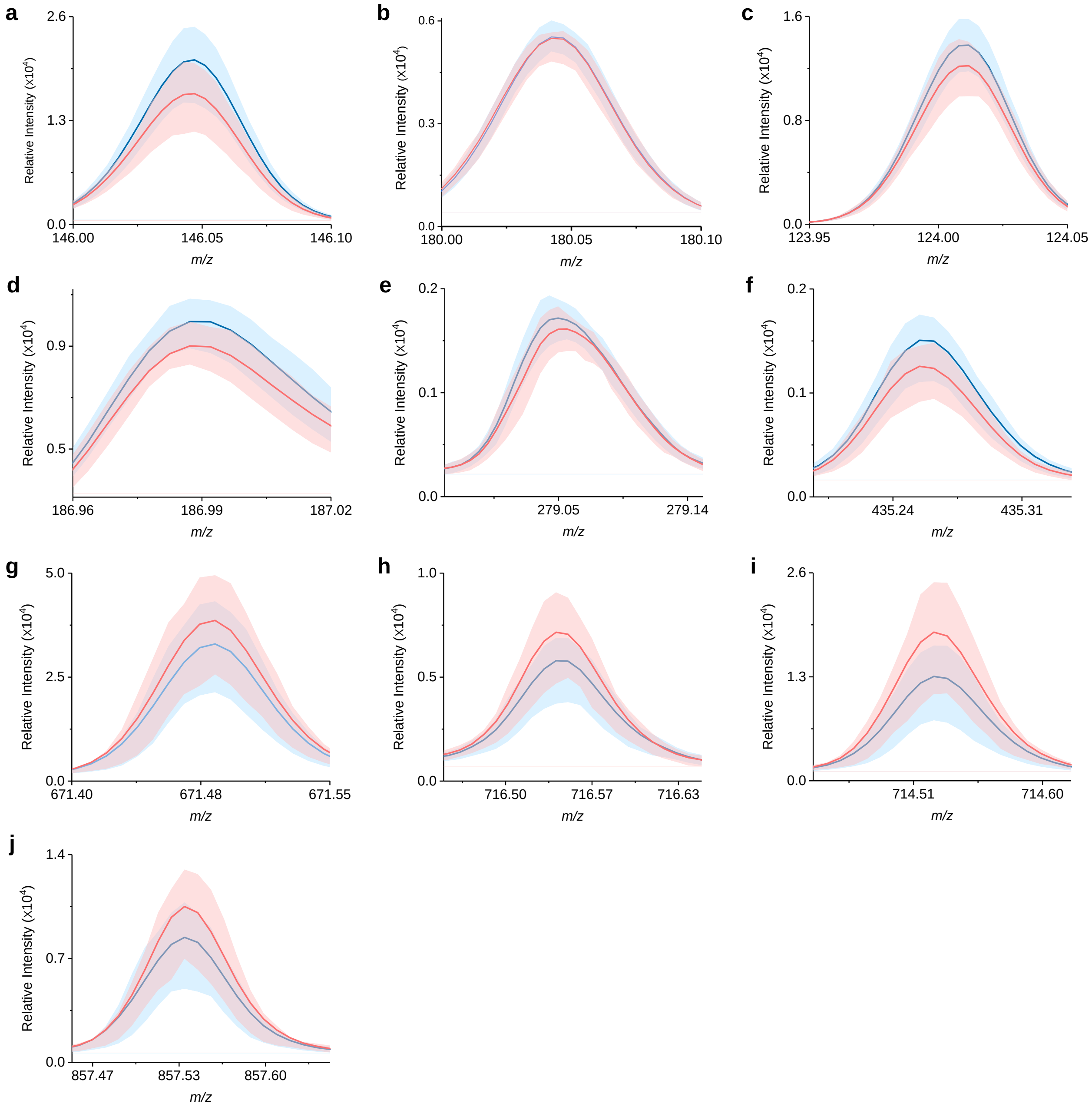

Figure S6. Intensity Comparison of the ten biomarkers between the healthy control and asymptomatic COVID-19 groups. a, glutamic acid; **b**, tyrosine (outlier removed); **c**, taurine; **d**, xanthine; **e**, uridine; **f**, LPA 18:1; **g**, PA 34:2 (16:0/18:2); **h**, PE 34:1 (16:0/18:1); **i**, PE 34:2 (16:0/18:2); **j**, PI 36:4 (16:0/20:4). Data are presented as mean values (line) ± IQR (shadow). The blue line and shadow derived from the samples of healthy control, and the red lines and shadows derived from the samples of asymptomatic COVID-19 patients.

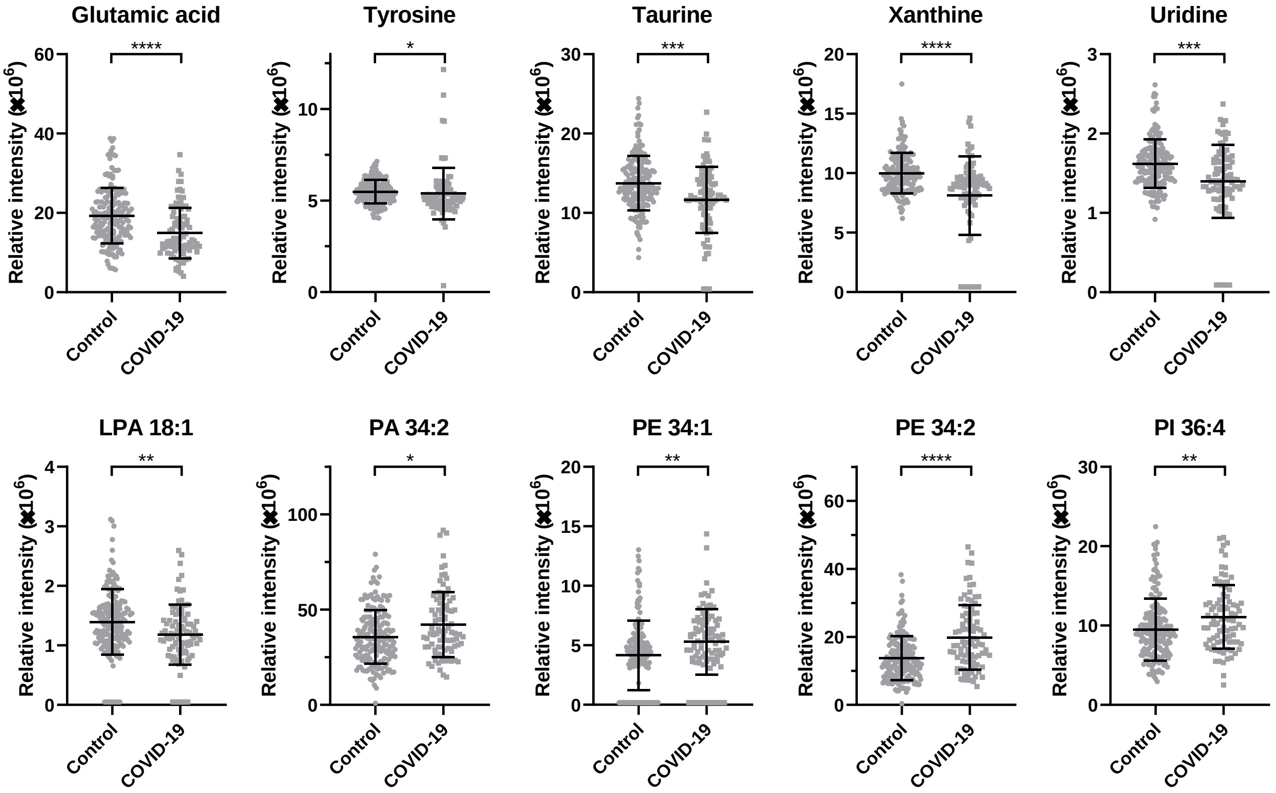

Figure S7. Comparison of the relative peak intensities of the ten biomarkers between the healthy control and asymptomatic COVID-19 patients. The metabolites and lipids are glutamic acid (p = 7.66E-6), tyrosine (p = 0.012), taurine (p = 7.31E-4), xanthine (p = 8.14E-6), uridine (p = 3.68E-4), LPA 18:1 (p = 0.0019), PA 34:2 (16:0/18:2) (p = 0.014), PE 34:1 (16:0/18:1) (p = 0.0018), PE 34:2 (16:0/18:2) (p = 4.28E-6), and PI 36:4 (16:0/20:4) (p = 0.0022). P values were calculated by Two-tailed Wilcoxon rank sum test with BH corrections (* $p < 0.05$; ** $p < 0.01$; *** $p < 0.001$; **** $p < 0.0001$). The outliers in each group were removed based on 3σ-rule. Error bars show mean ± SD.

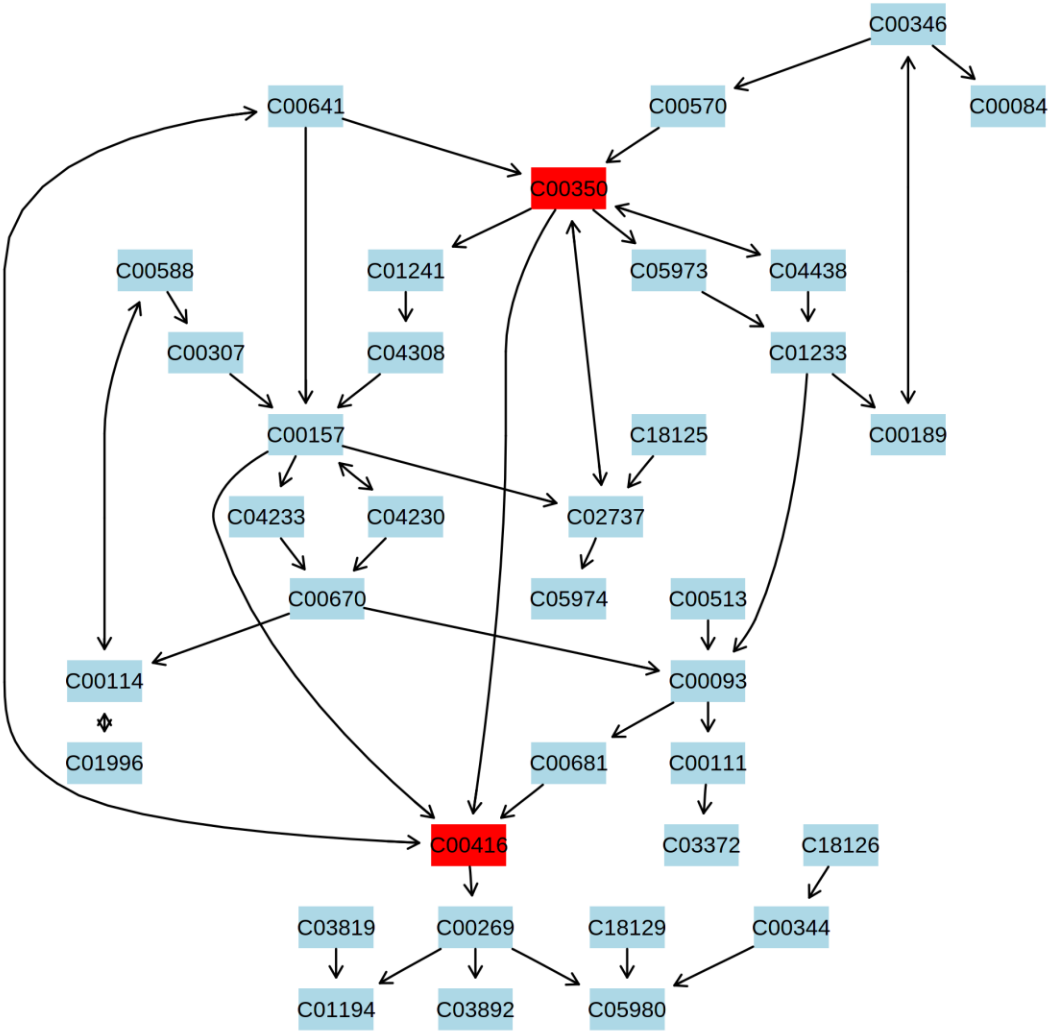

Figure S8. Schematic illustration of PE and PA metabolism. The compounds C00350 and C00416 represent phosphatidylethanolamine (PE) and phosphatidic acid (PA) that were in the data and used in topology analysis, respectively. The compounds with blue color represent metabolites that were not in the data and used as background in topology analysis. This plot was generated in MetaboAnalyst (https://www.metaboanalyst.ca/).

Table S1. Optimized hyperparameters of five machine learning models built with 219 features in five-fold nested cross-validation

ML Algorithms	Hyperparameters	Optimized values				
		Fold 1	Fold 2	Fold 3	Fold 4	Fold 5
KNN	n_neighbors	14	18	18	19	19
	weights	distance	distance	distance	distance	distance
RF	n_estimators	600	400	800	400	600
	max_depth	8	6	8	15	8
SVM	C	104	26	34	20	156
	gamma	0.00004	0.00025	0.00024	0.00037	0.00002
MLP	Hidden layer1	79	79	79	79	79
	Hidden layer2	80	79	80	80	81
	Hidden layer3	50	50	50	50	50
XGB	max_depth	3	2	2	2	3
	Learning_rate	0.15	0.15	0.15	0.15	0.15

Table S2. Performance metrics of five separate machine learning models with 219 features

Performance metrics	SVM	XGB	MLP	RF	KNN
Accuracy	0.891±0.023	0.865±0.032	0.847±0.036	0.799±0.039	0.730±0.019
Sensitivity	0.740±0.058	0.706±0.084	0.696±0.064	0.480±0.098	0.261±0.080
Specificity	0.967±0.040	0.945±0.018	0.923±0.047	0.961±0.028	0.967±0.026

Table S3. Optimized hyperparameters of five machine learning models built with 97 features in five-fold nested cross-validation

ML Algorithms	Hyperparameters	Optimized values				
		Fold 1	Fold 2	Fold 3	Fold 4	Fold 5
KNN	n_neighbors	19	15	11	19	13
	weights	distance	distance	distance	distance	distance
RF	n_estimators	400	800	400	1000	400
	max_depth	10	8	8	8	15
SVM	C	179	19	17	21	7
	gamma	0.00007	0.00027	0.00022	0.00018	0.00002
MLP	Hidden layer1	300	300	300	300	300
	Hidden layer2	45	45	45	45	45
	Hidden layer3	40	40	40	40	40
XGB	max_depth	2	2	2	2	3
	Learning_rate	0.3	0.15	0.15	0.2	0.2

Table S4. Performance metrics of five separate machine learning models with 97 features

Performance metrics	SVM	XGB	MLP	RF	KNN
Accuracy	0.916±0.025	0.891±0.040	0.908±0.026	0.854±0.046	0.770±0.014
Sensitivity	0.826±0.062	0.760±0.113	0.827±0.050	0.643±0.112	0.381±0.065
Specificity	0.962±0.028	0.956±0.014	0.950±0.027	0.961±0.028	0.967±0.052

Table S5. **Performance metrics of four stacking ensemble models with meta-learner**

Performance metrics	SVM/MLP	XGB/MLP	SVM/XGB	SVM/XGB/MLP
Accuracy	0.909±0.026	0.909±0.026	0.891±0.040	0.931±0.033
Sensitivity	0.827±0.050	0.827±0.050	0.760±0.113	0.891±0.069
Specificity	0.950±0.027	0.950±0.027	0.956±0.014	0.950±0.032
ROC_AUC	0.948±0.038	0.947±0.031	0.942±0.030	0.957±0.023
PR_AUC	0.879±0.093	0.896±0.068	0.893±0.069	0.910±0.075

Table S6. Performance indicators of four new ensemble models with novel voting algorithm

Performance metrics	SVM/MLP	XGB/MLP	SVM/XGB	SVM/XGB/MLP
Accuracy	0.934±0.027	0.931±0.035	0.931±0.029	0.934±0.029
Sensitivity	0.913±0.042	0.923±0.056	0.913±0.065	0.946±0.033
Specificity	0.945±0.034	0.934±0.028	0.940±0.032	0.929±0.037

Table S7. Ten metabolites and lipids biomarkers to distinguish asymptomatic COVID-19

Metabolites	Formula	Monoisotopic mass	Detected ion	Wilcoxon adjusted *p* value
Glutamic acid	$C_5H_9NO_4$	147.0532	$[M-H]^-$	7.66E−6
Tyrosine	$C_9H_{11}NO_3$	181.0738	$[M-H]^-$	0.012
Taurine	$C_2H_7NO_3S$	125.0147	$[M-H]^-$	7.31E−4
Xanthine	$C_5H_4N_4O_2$	152.0334	$[M+Cl]^-$	8.14E−6
Uridine	$C_9H_{12}N_2O_6$	244.0695	$[M+Cl]^-$	3.68E−4
LPA 18:1	$C_{21}H_{41}O_7P$	436.259	$[M-H]^-$	0.0019
PA 34:2 (16:0/18:2)	$C_{37}H_{69}O_8P$	672.473	$[M-H]^-$	0.014
PE 34:1 (16:0/18:1)	$C_{39}H_{76}NO_8P$	717.5309	$[M-H]^-$	0.0018
PE 34:2 (16:0/18:2)	$C_{39}H_{74}NO_8P$	715.5152	$[M-H]^-$	4.28E−6
PI 36:4 (16:0/20:4)	$C_{45}H_{79}O_{13}P$	858.5258	$[M-H]^-$	0.0022

Table S8. Optimized hyperparameters of three machine learning models built with 10 features in five-fold nested cross-validation

ML Algorithms	Hyperparameters	Optimized values				
		Fold 1	Fold 2	Fold 3	Fold 4	Fold 5
SVM	C	290	248	294	110	118
	gamma	0.00598	0.00596	0.00598	0.00378	0.00512
MLP	Hidden layer1	400	300	300	300	300
	Hidden layer2	200	300	300	300	300
	Hidden layer3	110	100	90	90	90
XGB	max_depth	2	4	2	4	3
	Learning_rate	0.01	0.03	0.01	0.01	0.01

Table S9. Performance indicators of four models built with 10 features

Performance metrics	SVM	MLP	XGB	SVM/XGB/MLP
Accuracy	0.796±0.047	0.803±0.031	0.799±0.033	0.821±0.038
Sensitivity	0.524±0.141	0.608±0.101	0.642±0.049	0.837±0.048
Specificity	0.934±0.013	0.901±0.033	0.879±0.055	0.814±0.059
ROC_AUC	0.834±0.048	0.806±0.049	0.820±0.035	0.850±0.026
PR_AUC	0.729±0.052	0.741±0.069	0.738±0.022	0.755±0.026
